# The aromatic amino acid hydroxylase genes *AAH1* and *AAH2* in *Toxoplasma gondii* contribute to transmission in the cat

**DOI:** 10.1371/journal.ppat.1006272

**Published:** 2017-03-13

**Authors:** Zi T. Wang, Shiv K. Verma, Jitender P. Dubey, L. David Sibley

**Affiliations:** 1 Department of Molecular Microbiology, Washington University School of Medicine, St. Louis, Missouri, United States of America; 2 United States Department of Agriculture, Agricultural Research Service, Beltsville Agricultural Research Center, Animal Parasitic Diseases Laboratory, Beltsville, Maryland, United States of America; University of Wisconsin Medical School, UNITED STATES

## Abstract

The *Toxoplasma gondii* genome contains two aromatic amino acid hydroxylase genes, *AAH1* and *AAH2* encode proteins that produce L-DOPA, which can serve as a precursor of catecholamine neurotransmitters. It has been suggested that this pathway elevates host dopamine levels thus making infected rodents less fearful of their definitive Felidae hosts. However, L-DOPA is also a structural precursor of melanins, secondary quinones, and dityrosine protein crosslinks, which are produced by many species. For example, dityrosine crosslinks are abundant in the oocyst walls of *Eimeria* and *T*. *gondii*, although their structural role has not been demonstrated, Here, we investigated the biology of *AAH* knockout parasites in the sexual reproductive cycle within cats. We found that ablation of the *AAH* genes resulted in reduced infection in the cat, lower oocyst yields, and decreased rates of sporulation. Our findings suggest that the *AAH* genes play a predominant role during infection in the gut of the definitive feline host.

## Author summary

*Toxoplasma gondii* is an intracellular parasite that infects up to one-quarter of humans worldwide. Although it can infect virtually any warm-blooded animal, its definitive host is the cat where the sexual cycle occurs in enterocytes of the small intestine, producing microscopic, durable oocysts that are shed in feces and can remain infectious for extended periods of time in the environment. Two parasite genes, *AAH1* and *AAH2*, code for aromatic amino acid hydroxylases, which produce L-DOPA, the precursor to dopamine. However, L-DOPA is also a precursor of other structural molecules including dityrosine, which may play a role in the wall of the oocyst. We investigated the effect of *AAH* deletion on the ability of the parasites to undergo sexual reproduction in cats, and found that *AAH*-deficient parasites were defective in their ability to produce oocysts, and those oocysts were partially defective in their ability to undergo maturation once produced. Collectively, these results suggest that the *AAH* genes play their primary role in transmission through the definitive host.

## Introduction

*Toxoplasma gondii* is an obligate intracellular parasite and a member of the phylum Apicomplexa. It is related to *Plasmodium* spp., the causative agents of malaria, as well as parasites of human and veterinary importance including *Cryptosporidium* spp., *Eimeria* spp., and *Neospora* spp. *T*. *gondii* is one of the most widely distributed parasites in the world, and can be found on every continent and in virtually every species of warm-blooded animal investigated [1]. The definitive host of *T*. *gondii* is the cat, including all members of the family Felidae [2]. Within enterocytes of the cat intestine, *T*. *gondii* is capable of producing oocysts that are shed in the feces [3]. Oocysts are spheroid, 10–12 μm in size, and are comprised of an outer wall encapsulating two sporocysts that each contain four infectious sporozoites [4]. Oocysts are structurally robust with an elasticity and strength similar to common plastics [5]. They are very environmentally resilient, able to withstand a wide range of physical and chemical challenges including bleach, ethanol, acids, and bases [6], can stay infectious for years in the environment [7], and represent a significant source of dissemination for the parasite [8]. Omnivorous and herbivorous animals such as livestock can become infected by eating oocysts that contaminate rangeland, or by ingestion of contaminated water supplies [1]. Humans can also be infected by accidental ingestion of oocysts in contaminated food sources such as vegetables [9], or by ingestion of oocysts in water [10].

The walls of *T*. *gondii* oocysts are highly proteinaceous, composed of >90% protein [6], as well as β 1–3 glucan carbohydrates [11], and acid-fast lipids [12]. Large-scale proteomic analyses have identified 1,031 [13] or 1,304 [14] individual, non-redundant proteins associated with the oocyst. Although the function and localization of many remain unknown, two classes of oocyst wall structural proteins have been identified in other apicomplexans. In *Cryptosporidium parvum*, cysteine-rich *COWP*s (*Cryptosporidium* oocyst wall proteins) form a proteinaceous structure through extensive disulfide bridges [15]. Alternatively, tyrosine-rich *EmGam* (*Eimeria* gametocyte*)* proteins form a proteinaceous structure through extensive dityrosine linkages in the oocyst walls of *Eimeria maxima* [16–18]. The *T*. *gondii* genome contains seven cysteine-rich *TgOWP* proteins that are thought to be homologous to the *COWPs*. *TgOWP* proteins *TgOWP1-3*, were characterized and described in the outer oocyst walls but not the inner sporocyst walls [19]. Although *T*. *gondii* does not contain clear homologues of *Eimeria*’s *EmGam* proteins, many tyrosine-rich proteins have been identified in both outer oocyst wall and inner sporocyst wall fractions by mass spectrometry [13, 14], although they have not been definitively identified as structural components in the oocyst wall [5].

The genome of *T*. *gondii* contains two genes encoding aromatic amino acid hydroxylases referred to as *AAH1* and *AAH2* [20]. These genes encode predicted secretory proteins that catalyze conversion of phenylalanine to tyrosine, and tyrosine to 3,4 dihydroxyphenylalanine (L- DOPA) [20]. Conversion of tyrosine to L-DOPA is the rate-limiting step of dopamine synthesis in metazoans [21]. Although initial studies suggested that these enzymes are involved in modulating dopamine production in mammalian hosts [20, 22, 23], we were unable to replicate these findings in our previous work that focused on generating a knockout of *AHH2* [24]. Moreover, our findings failed to reveal an elevated level of dopamine in chronically infected animals or in dopaminergic cells infected *in vitro* [24], consistent with recent reports by other authors [25, 26]. Hence, we sought to investigate other pathways that could require aromatic amino acid hydroxylase activity by *T*. *gondii*.

L-DOPA serves as a precursor to many structural components across other branches of eukaryotes, including helminths, molluscs, annelids, ascidians [27], and insects [28], and in coccidian apicomplexan parasites. In *E*. *maxima*, L-DOPA has been identified in the oocyst, where conversion of tyrosine to 3,4 dihydroxyphenylalanine (i.e. L-DOPA) on the tyrosine-rich *EmGam* precursor glycoproteins is an intermediate step in the formation of dityrosine crosslinks that provide structural strength to the *Eimeria* oocyst wall [16, 17]. Dityrosine has a strong blue auto fluorescence under UV light, a fluorescence observed in the oocysts of both *Eimeria* and *T*. *gondii* [29]. Furthermore, microarray data indicates that the *AAH* genes are upregulated during oocyst development in *T*. *gondii* [30], and protein mass spectrometry identifies both tyrosine-rich proteins and the tyrosine hydroxylases AAH1 and AAH2 in the oocyst of *T*. *gondii* [13, 14]. In contrast, these hydroxylases are not found in similar mass spectrometric analyses of tachyzoites or bradyzoites [14].

Here we sought to investigate the role of the *T*. *gondii AAH* genes in oocyst development using a combination of genetic, cellular, and biochemical studies. Although deletion of *AAH2* alone caused a mild defect, ablation of *AAH1*, or loss of both genes, caused a severe defect in infection of the intestine and oocyst yield. Together, our results show that the *AAH* genes play an important role in parasite development during the sexual cycle in the intestinal epithelium of the cat.

## Materials and methods

### Ethics statement

Animal studies on mice were approved by the Institutional Animal Studies Committee (School of Medicine, Washington University in St. Louis). All procedures on cats were carried out in accordance with relevant guidelines and regulations following a protocol approved by the Beltsville Area Animal Care and Use Committee (BAACUC), United States Department of Agriculture, Beltsville, MD, USA.

### Parasite strains

Parasites were propagated by serial passage in human foreskin fibroblast (HFF (obtained from the laboratory of Dr. John Boothroyd, Stanford University School of Medicine)) cells grown in Dulbecco’s Modified Eagle Medium (DMEM) (Life Technologies, Carlsbad, CA) containing 10% fetal bovine serum (FBS) (Hyclone, Logan, UT) 10mM HEPES, pH 7.4, 1mM glutamine, 10 μg/mL gentamycin, under 5% CO_2_ at 37°C (D10 media). The parental ME49*Δhxg*::*Luc* strain was obtained from Laura Knoll (University of Wisconsin, Madison) [31]. A complete list of strains and clones used or generated in this study is provided in S1 Table. Tachyzoites were maintained by serial passage in HFF cells, grown as above. For induction of bradyzoites, cultures were switched to Roswell Park Memorial Institute 1640 medium (RPMI 1640), 50 mM HEPES pH 8.2 (Thermo Fisher Scientific, Grand Island, NY) and grown at 37°C without CO_2_, as described previously [32]. Cultures were determined to be free of mycoplasma using the e-Myco plus kit (iNtron Biotechnology). Parasites were harvested for experiments by scraping infected HFF monolayers into suspension, lysing HFFs and liberating tachyzoites by passage through a 20 *g* needle, and purifying tachyzoites with a 3.0 micron polycarbonate filter.

### CNV estimation

CNV data was obtained from an Illumina sequencing dataset of sixteen *T*. *gondii* reference strains and 46 non-reference strains, aligned using Bowtie2 using the end-to-end option [40].

### Generation of plasmids

A complete list of plasmids used or generated in this study is provided in S2 Table. CRISPR/Cas9 plasmids were adapted from the *pSAG1*:*CAS9*,*U6*:*sgUPRT* plasmid previously generated by our lab [33]. The guide RNA of the plasmid was modified to target the *AAH2* 5’ UTR by Q5 mutagenesis (New England Biolabs, Ipswich, MA), creating the plasmid *pSAG1*:*CAS9*,*U6*:*sgAAH2*. A second guide RNA expression cassette targeting the *AAH2* 3’ UTR was inserted into the same plasmid backbone by traditional cloning steps to create the CRISPR/Cas9 *AAH2* double cut plasmid *pSAG1*:*CAS9*,*U6*:*dgAAH2*. The same plasmid backbone was similarly adapted to target the *AAH1* 5’ and 3’ UTRs (*pSAG1*:*CAS9*,*U6*:*sgAAH1* and *pSAG1*:*CAS9*,*U6*:*dgAAH1*). The *pSAG1*:*CAS9*,*U6*:*sgUPRT* plasmid described previously [33] was also modified to create a double-cutting CRISPR/Cas9 plasmid targeting the *HXGPRT* gene *pSAG1*:*CAS9*,*U6*:*dgHXGPRT* [34]. Plasmids used to generate the Δ*aah2* knockout using the *HXGPRT* selectable marker to replace the gene in the ME49*Δhxg*::*Luc* strain [31], and to restore expression of *AAH2* were described previously [24]. Plasmids used to generate the Δ*aah1* mutant by replacement with the selectable marker *DHFR-Ts*, and to complement expression with a cDNA construct targeted to the uracil phosphoribosyl transferase (*UPRT*) locus, were created using Gibson assembly (New England Biolabs).

### Generation of parasite transgenic lines

To generate transgenic ME49 parasites, 10^7^ tachyzoites, harvested as described above, were mixed with 5μg of CRISPR plasmids and 15μg of the appropriate homologous repair construct as plasmids linearized by restriction digest. Parasites were transfected by electroporation, and allowed to recover on HFF monolayers for 24 h. Positive selection for the *HXGPRT* cassette was done with 25 μg/mL mycophenolic acid (Sigma-Aldrich, St. Louis, MO) supplemented with 50 μg/mL xanthine (Sigma-Aldrich) [34]. Negative selection against the HXGPRT cassette was done with 340 μg/mL 6-Thioxanthine (Toronto Research Chemicals, Toronto, ON) [35]. Positive selection for the *DHFR-Ts* construct was done with 5μM pyrimethamine (Sigma-Aldrich) [36]. Negative selection against the *UPRT* locus was done with 10μM 5-fluorodeoxyuracil (FUDR) (Sigma-Aldrich) [37]. Clones were isolated by limiting dilution in 96-well plates containing HFF monolayers, grown as above. Clones were screened by PCR against the selectable marker and the *AAH* genes (S3 Table).

### Growth assays

Parasites were seeded into T-25s containing monolayers of HFF cells and allowed to invade and grow for 24 h. Infected T-25s were then rinsed three times with PBS to remove any extracellular parasites. Intracellular parasites were harvested as previously described, counted by hemocytometer and seeded into 96-well plates containing monolayers of HFFs with fresh D10 media at a concentration of 10^5^ parasites per well. Plates were allowed to grow for 24 h before being lysed with 30uL of 1x Cell Culture Lysis Reagent (Promega, Madison, WI). Luminescence was developed with the Luciferase Assay Kit (Promega), and imaged on a Cytation 3 imaging system (Biotek, Winooski, VT).

### Infection of mice

Parasites were harvested as previously described, counted by hemocytometer, and diluted into PBS. Eight-week old female CD1 mice (Charles River Laboratories, Wilmington, MA) were injected i.p. in a volume of 200 μL PBS containing 10^3^ parasites and monitored daily. One month post-infection, mice were euthanized by CO_2_ asphyxiation followed by cervical dislocation. Brains were removed, homogenized by passage through a 20 *g* needle, and stained with *Dolicos biflorus* lectin (DBL) as previously described [38]. Fifteen μL of stained homogenate was examined using a Zeiss wide-field epifluorescence microscope. Three separate aliquots were counted per brain sample, and total brain cyst load was determined based on the total volume of the brain homogenate and the average count per 15 μL.

### *Toxoplasma gondii* infection in cats

All procedures described here were carried out in accordance with relevant guidelines and regulations following a protocol approved by the Beltsville Area Animal Care and Use Committee (BAACUC), United States Department of Agriculture, Beltsville, MD, USA. *T*. *gondii* -free kittens (10- to 12-week old) were used to study *T*. *gondii* infections. Briefly, *T*. *gondii* infected mouse brains were homogenized by syringe and fed to the cats by placing them at the back of the tongue. All feces for each cat were collected daily after feeding infected mouse brains, and examined for *T*. *gondii* oocysts. The screening and harvesting of *T*. *gondii* oocysts were done between 3 to 21 days after infection by following procedures as described previously [1]. Cats were euthanized on day 21 post infection and blood was collected to do modified agglutination tests (MAT) to test for immunological reactivity to *T*. *gondii* antigens. Oocysts were collected by floatation methods using sucrose solution with a specific gravity of 1.15 or higher. Concentrated oocyst pellets were suspended in an aqueous solution containing 2% H_2_SO_4_, and aerated on the shaker for 7 days at room temperature (20–22°C) to allow for oocyst sporulation. Oocysts were counted using a disposable hemocytometer. Total oocysts shed by individual cats were calculated based on total counts, dilution factor, and total volume.

### Histological examination

For histological studies, infected cats were euthanized at day 6/7 and portions of intestinal ileum were fixed in 10% buffered neutral formalin. Fixed tissues were cut into sections (2.5 x 0.7 cm), placed in cassettes, embedded in paraffin, and sectioned 4–5 μm thick. Slides were deparaffinized, rehydrated, and stained with hematoxylin and eosin (Leica Microsystems, Buffalo Grove, IL), or by immunohistochemistry with Rabbit anti-RH polyclonal antibody [39] and Streptavidin-HRP (Jackson Labs, West Grove, PA), according to standard protocols [1].

### Microscopy

Images were taken on a Zeiss AxioSkop wide field epifluorescence microscope equipped with AxioCam CCD camera and images were captured using AxioVision v3.1 (Carl Zeiss Inc., Thornwood, NY). For each image, 10 μL of oocyst-laden cat fecal suspension were placed on a slide and imaged with a DAPI filter (300–390 nm excitation, 420 nm emission).

### Statistics

Statistical analysis was done in Prism 6 for Mac OSX (GraphPad Software, La Jolla, CA). One-way and two-way ANOVAs for parametric data sets and Kruskal-Wallis tests for nonparametric data sets were conducted with a threshold of *P* ≤ 0.05 considered significant.

## Results

### The ME49 strain has a duplication in the *AAH2* gene

**Fig 1:**
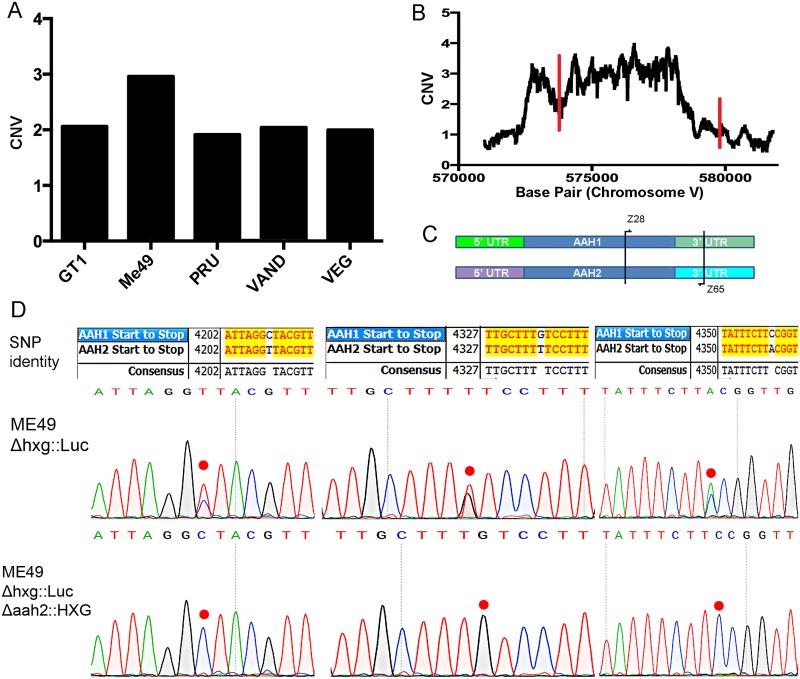
Copy number analysis of *AAH2* in *T*. *gondii* strains. (A) Copy number variation (CNV) of Tg_ME49_212740 (*AAH2*) in five representative strains. (B) CNV at each base across the *AAH2* gene in ME49. Red lines indicate the start and stop codons of *AAH2*. (C) PCR using primers (Z28+ Z65) (S3 Table) common to both *AAH1* and *AAH2* were used against the ME49 genome to amplify both genes for Sanger sequencing and single nucleotide polymorphism (SNP) determination. (D) Sanger sequencing of PCR fragments against the *AAH* genes of the wild type ME49 *Δhxg*::*Luc* strain shows a 2:1 ratio of *AAH2* to *AAH1* SNPs, indicating a duplication of the *AAH2* gene. Each SNP in the chromatograph is marked by a red dot. In the *Δaah2*::*HXG* knockout, all *AAH2* SNPs are no longer visible, indicating loss of both copies of the *AAH2* gene.

Previous studies have described two genes *AAH1* and *AAH2* that are very closely related and located on chromosome V (ToxoDB ver. 8 ME49 genome) [40]. Analysis of copy number variation (CNV) of *AAH2* TgME49_212740 showed approximately two copies in the type 1 strain GT1, the type 2 strain Pru, the type 3 strain VEG and the type 10 strain VAND (Fig 1A). In contrast, the type 2 strain ME49 had a CNV level consistent with three copies (Fig 1A). Although *AAH1* and *AAH2* genes appeared as tandem loci in ToxoDBv8, v9 and subsequent assemblies placed *AAH1* (TgME49_087510) on an unassembled contig (KE139705), and contains an additional third gene consistent with *AAH2* (TgME49_212710) on another unassembled contig (KE139818), while recognizing only one tyrosine hydroxylase *AAH2* within the parasite genome itself, located on chromosome V.

Mapping reads across each base pair of the *AAH2* locus showed a consistent CNV of approximately 3 across the coding region of *AAH2* (Fig 1B). To further examine the nature of the predicted third copy, we amplified the 3’ region of *AAH1/ AAH2* using primers common to both genes (Fig 1C). We then interrogated the nature of the alleles present in the ME49 strain using Sanger sequencing. Inspection of the chromatographs from Sanger sequencing indicated a 2:1 ratio of *AAH2* to *AAH1* single nucleotide polymorphisms (SNPs), consistent with a duplication of *AAH2* in ME49 (Fig 1D). These sequencing results also confirmed the ToxoDB ver. 8 arrangement of flanking regions for *AAH1* and *AAH2*.

### Deletion of *AAH1* and *AAH2* in the ME49*Δhxg*::*Luc*

**Fig 2:**
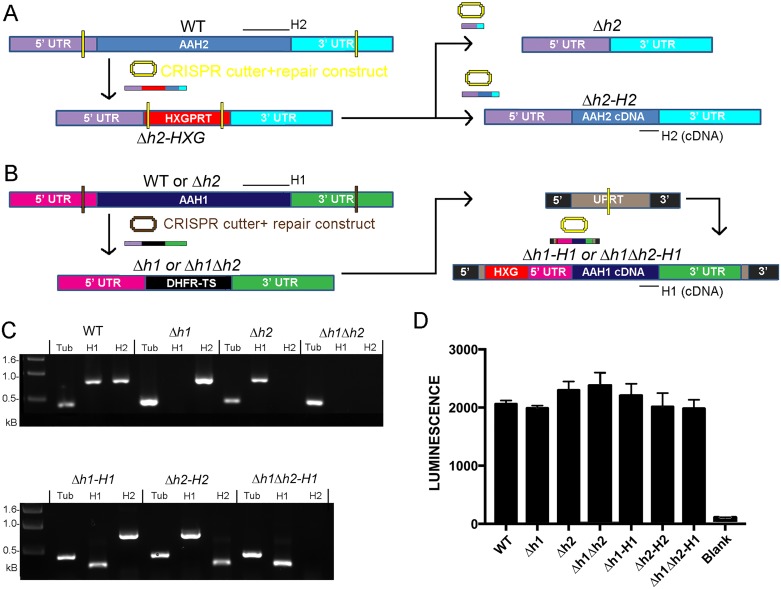
Disruption of the *AAH1* and *AAH2* genes. (A) Schematic of the *AAH2* knockout strategy in the wild-type ME49 *Δhxg*::*Luc* strain (referred to as WT). A CRISPR-Cas9 construct with guide RNAs targeted to the 5’ and 3’ UTRs of *AAH2* was co-transfected with the *pΔaah2*::*HXG* plasmid (S2 Table) and selected for with MPA +Xanthine to delete *AAH2* to produce the clone *Δaah2*::*HXG* (*Δh2-HXG*). Subsequently, the *HXG* gene was replaced with either a clean fusion of the *AAH2* 5’ and 3’ UTRs (*pΔaah2*) or an *AAH2* cDNA construct (*pAAH2*). (S2 Table) using 6-thioxanthine selection against the *HXG* locus to create the clean knockout clone *Δaah2* (*Δh2*) (upper panel) and the complement clone *Δaah2*::*AAH2* (*Δh2-H2*) (lower panel). Yellow Bars: CRISPR targeting sites. Black bars: PCR screening primer target regions (S3 Table). (B) Schematic of the knockout strategy for *AAH1*. A CRISPR-Cas9 construct with guide RNAs targeted to the 5’ and 3’ UTRs of *AAH1* was co-transfected with the *pΔaah1*::*DHFR-Ts* repair construct (S2 Table) into WT or *Δaah2* parasites to create the clones *Δaah1* (*Δh1*) and *Δaah1Δaah2* (*Δh1Δh2*). Transfectants were selected for via pyrimethamine resistance. Subsequently, using *pΔuprt*::*AAH1*::*HXG*, a cDNA copy of *AAH1* driven by its native 5’ and 3’ UTRs was complemented into the *UPRT* locus by means of the *HXGPRT* drug resistance marker selected for with MPA +Xanthine, negative selection against *UPRT* with FUDR, and a single-cutting CRISPR-Cas9 construct targeted to the *UPRT* gene (S2 Table), creating the complement clones *Δaah1-AAH1* (*Δh1-H1*) and *Δaah1Δaah2-AAH1* (*Δh1Δh2-H1*). Brown & Yellow Bars: CRISPR targeting sites. Black bars: PCR screening primer target regions (S3 Table). (C) PCR verification of successful ablation and complementation of knockouts. Expected product sizes: *Tubulin* (Tub): 0.378kb. *AAH1* (H1): 0.745kb (Native), 0.278kb (cDNA). *AAH2* (H2): 0.745kb (Native), 0.278kb (cDNA). (D) Growth assays of parasites seeded into 96-well plates and allowed to proliferate for 24 h, then quantified using a luciferase assay. The WT, *Δh1*, Δh2, *Δh1Δh2*, *Δh1-H1*, *Δh2-H2*, and *Δh1Δh2-H1* parasites showed no significant difference in total growth (Kruskal-Wallis test, *P* = 0.0672, N = 3 per strain).

We previously reported that deletion of *AAH2* in the type 2 Pru strain has no effect on growth *in vitro* or development of bradyzoites *in vivo* [24]. To examine the ability of *Δaah2* mutants to be passaged through cats, we decided to generate a similar *Δaah2* deletion in the type 2 ME49 strain, which has a high capacity for oocyst generation. We targeted the *AAH2* gene for replacement with the *HXGPRT* selectable marker in the ME49*Δhxg*::*Luc* strain (referred to as wild type (WT)), which has a deletion on the *hxgprt* locus and is also tagged with firefly luciferase. To efficiently delete the *AAH2* gene, a CRISPR/Cas9 plasmid containing two guide RNAs targeting the 5’ and 3’ UTRs of *AAH2* was created (Fig 2A) (S2 Table). This double-cutting plasmid was co-transfected into the parental WT strain with an *HXGPRT* drug resistance cassette targeted to the *AAH2* locus to create the clone *Δaah2*::*HXG* (S1 Table). Sanger sequencing of this clone revealed that both copies of *AHH2* had been removed, while the *AHH1* gene remained intact (Fig 1D). To remove the *HXGPRT* selectable marker, a CRISPR/Cas9 double-cutter of *HXGPRT* was co-transfected with an *aah2*-null fusion construct of the *AAH2* 5’ and 3’ UTRs (*pΔaah2*) or a complement construct of its 5’ and 3’ UTRs appended to a cDNA copy of *AAH2* (*pAAH2*) to make the clean knockout clone *Δaah2* (referred to as *Δh2*) and the complement clone *Δaah2*::*AAH2* (referred to as *Δh2-H2*), which restores expression of AAH2 (Fig 2A).

Subsequently, to knock out *AAH1*, we created a double-cutting CRISPR/Cas9 construct targeted to the UTRs of the *AAH1* gene, and co-transfected it with a *Δaah1*::*DHFR-Ts* construct (*pΔaah1*::*DHFR-Ts*) (S2 Table) into WT or *Δh2* strains to make the clones *Δaah1* (referred to as *Δh1*) and *Δaah1Δaah2* (referred to as *Δh1Δh2*) (Fig 2B) (S1 Table). To restore *AAH1*, we co-transfected the *pSAG1*:*CAS9*,*U6*:*sgUPRT* CRISPR plasmid with a repair construct containing *HXGPRT* and a cDNA copy of *AAH1* to create the clones *Δaah1-AAH1* (referred to as *Δh1-H1*) and *Δaah1Δaah2-AAH1* (referred to as *Δh1Δh2-H1*) (S1 Table).

### Growth and differentiation of the wild type and knockout strains

**Fig 3:**
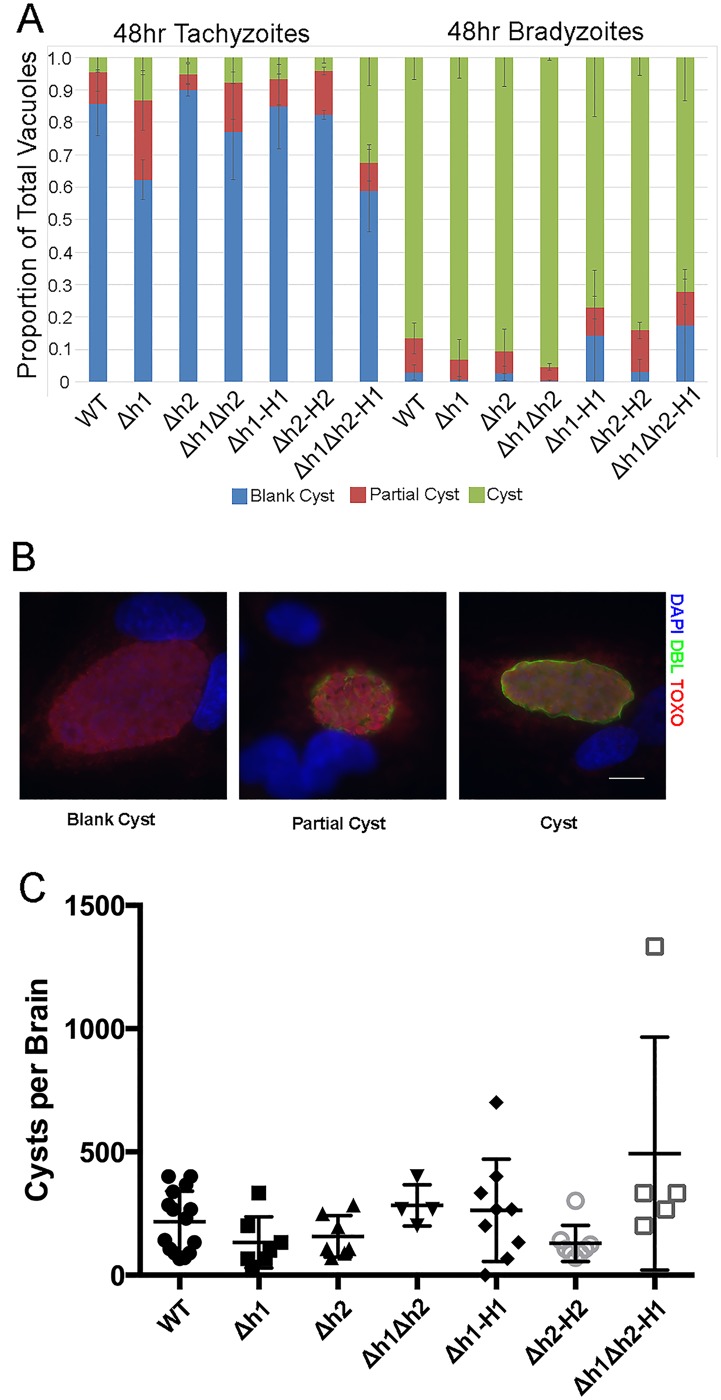
Development of bradyzoites *in vitro* and *in vivo*. (A) There was no significant difference in bradyzoite differentiation *in vitro* across the parasite lines in either tachyzoite conditions (left) (*P*> 0.99) or bradyzoite conditions (right) (*P*> 0.99) (Two-way ANOVA).(B) Representative pictures of tachyzoites, partial cysts, and complete cysts produced *in vitro* as assessed by DBL staining. (C) Brain cyst yields in mice 1–2 months post-infection. All parasite lines produced similar numbers of tissue cysts *in vivo* (Kruskal-Wallis, ns).

Having generated a single knockout of each of the Δ*aah1* and Δ*aah2*, as well as the double Δ*aah1*Δ*aah2* knockout and several complemented strains, we decided to compare their growth and differentiation abilities *in vitro* and *in vivo*. Consistent with the fact that we were able to obtain the mutants readily in culture without any apparent growth defect, their growth as tachyzoites was similar when compared using a highly quantitative luciferase assay (Fig 2D). We also tested their ability to differentiate to bradyzoites *in vitro* under conditions of pH 8.2 stress, as assessed by staining with *Dolichos biflorus* lectin, which stains carbohydrates in the cyst wall. We observed that the ability of the knockout and complemented strains to differentiate into bradyzoites was unaffected (Fig 3A and 3B). Additionally, these strains were injected into mice in order to assess their ability to form cysts in the brains of chronically infected mice. Loss of the *AAH1* or *AAH2* genes did not affect the ability to produce cysts in the mouse brain, and although the complementation of the double Δ*aah1*Δ*aah2* (*Δh1Δh2*) with the *AAH1* gene showed slightly higher cyst burdens, this was not significant (Fig 3C). The lack of a discernable phenotype on the development of bradyzoites is consistent with our previous studies in the Pru strain, albeit this was previously only tested with the Δ*aah2* mutant [24].

### Deletion of *AAH1/ AAH2* causes a defect in oocyst production and development

**Fig 4:**
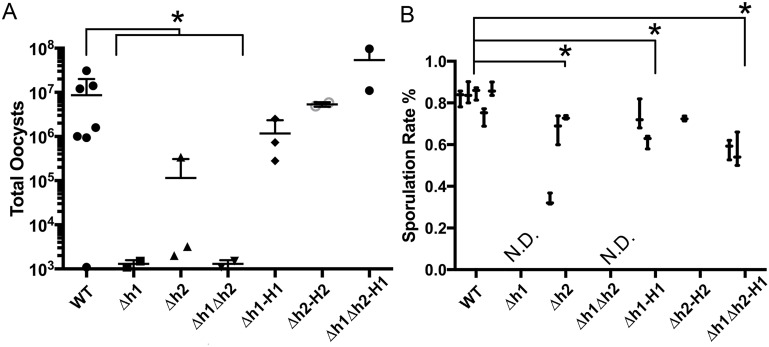
Development of oocysts following infection of cats. (A) Yields of oocysts shed from infected cats. The yield of knockout mutants as a whole were significantly reduced relative to the wild-type (Kruskal-Wallis, *P* ≤0.05), however due to low sample size, pairwise comparisons between each mutant and the WT approached, but did not reach significance (*Δh1 P* = 0.116, *Δh2 P* = 0.821, *Δh1Δh2 P* = 0.116). (B) The sporulation success rate of shed oocysts shows a significant defect in mutant lines (Dunn’s multiple comparisons test, for wild type vs. *Δh2 P* = 0.0008; and for the wild type vs. *Δh1-H1 P* = 0.0178 and *Δh1Δh2-H1 P*< 0.0001). The oocyst yields of *Δh1* and *Δh1Δh2* parasites were not sufficient to allow quantification (not done = N.D.). Each result is displayed as the Mean ±SD of three replicate counts of oocysts (n ≥ 50 per count) from one cat.

To investigate development during the sexual cycle, tissue cysts contained in mouse brain homogenate were fed orally to cats and oocyst shedding was monitored. The normal prepatent period for oocyst shedding following infection with bradyzoites is 3–5 days with peak shedding from 5–8 days [2]. Consistent with this, cats that showed oocysts shedding commenced within the first week. However, to be sure we collected all of the oocysts produced, we extended the observation period to 21 days. Infection with the WT strain consistently yielded around 10^6^−10^7^ total oocysts shed during this time period (Fig 4A). Although the Δ*aah2* (*Δh2*) mutant yielded much lower levels of oocyst in two of three cats, a third animal showed only ~ 10 fold reduction to ~10^5^ total oocysts (Fig 4A). In contrast, the *Δaah1* mutant (*Δh1*) and *Δaah1Δaah2* double mutant (*Δh1Δh2*) showed a severe defect in oocyst yield in two of two cats tested, leading to only ~10^3^ total oocysts per animal (Fig 4A). The differences observed in these animals were significant when the knockout strains were compared as a whole to the wild type (Fig 4A). However, they did not reach statistical significance when compared individually to the wild type (Fig 4A), due to the low sample sizes used. Given the magnitude of the phenotype, and the consistency among mutants, we did not feel it was worthwhile to use more animals simply to achieve an arbitrary level of statistical significance. The moderate defect in the Δ*aah2* (*Δh2*), and the very severe defect in both the Δ*aah1* (*Δh1*) and the double Δ*aah1Δaah2* (*Δh1Δh2*) knockouts, were fully restored in the respective complemented strains (Fig 4A).

**Fig 5:**
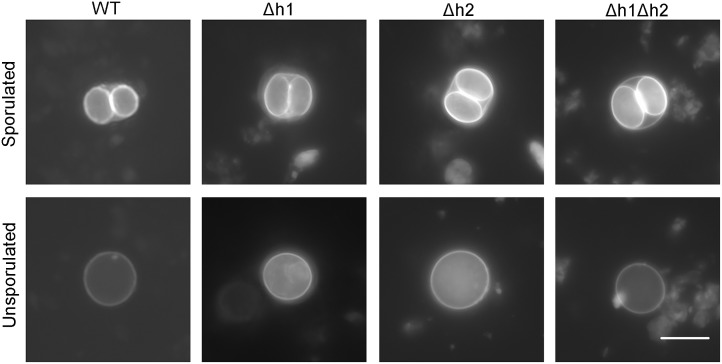
Representative fluorescence microscope images of dityrosine autofluorescence in sporulated and unsporulated oocysts of WT, *Δh1*, *Δh2*, and *Δh1Δh2* oocysts. All images were taken at 1000-1600ms exposure using a DAPI UV filter, but due to rapid photobleaching and differing levels of background signal in different oocyst fecal suspensions, direct comparison and quantification of fluorescence is not feasible. Scale bar = 10μm.

We also tested the ability of shed oocysts to undergo sporulation, since meiosis occurs after oocyst shedding. The sporulation rate is a measure of viability as unless oocyst mature to form sporozoites, they remain non-infectious [4]. Wild type oocysts showed a successful sporulation rate of 75–80% and this dropped significantly to ~ 60% in the *Δaah2* (*Δh2*) (Fig 4B). Oocyst shedding was so low that we were not able to adequately quantify the efficiency of sporulation in the single Δ*aah1* (*Δh1*) and double Δ*aah1*Δ*aah2* (*Δh1Δh2*) mutants (Fig 4B); however, based upon very limited counts, the sporulation success rate of these strains varied from 10–50% across samples. Complementation of *AAH1* to the *Δaah1* (*Δh1*) single knockout or the Δ*aah1*Δa*ah2* (*Δh1Δh2*) double knockout partially rescued sporulation efficiency (Fig 4B). Dityrosine fluorescence is normally much stronger on the inner sporocyst walls, and consequently the intensity of fluorescence under UV illumination was lower in unsporulated oocysts (Fig 5). Although the single and double mutants showed variable defects in the extent of sporulation (Fig 4B), when oocyst sporulation was normal, the resulting fluorescence of the inner sporocyst walls was similar among all the strains tested (Fig 5). We successfully hatched *Δaah2* oocysts and recovered them back into *in vitro* culture as tachyzoites, indicating that the oocysts that appeared to develop successfully were viable. However, the yield of the *Δaah1* and *Δaah1Δaah2* knockouts was too low to allow for this method of recovery.

**Fig 6:**
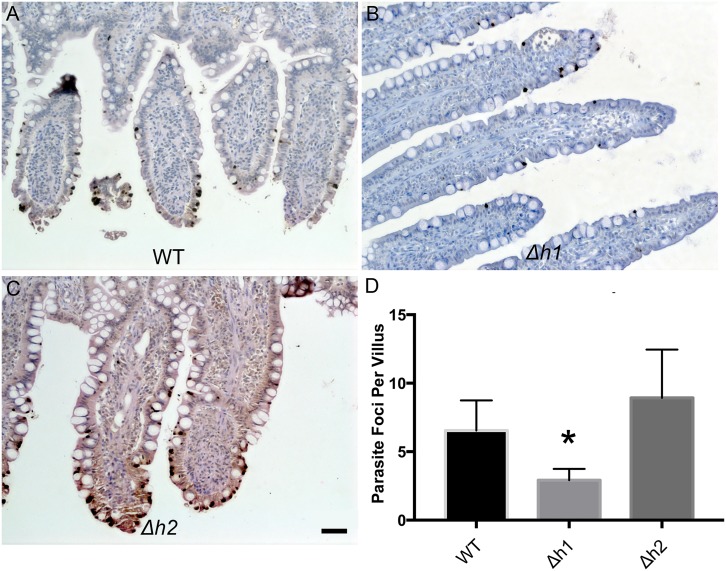
Representative images of cat intestinal ileum infected with WT (A), *Δh1* (B), and *Δh2* (C) parasites stained with a polyclonal anti- *T*. *gondii* antibody and Streptavidin-HRP (brown). Parasites are located throughout the intestinal villi (dark deposits). Scale bar = 50μm. (D) Parasite density per villus in intestinal sections infected with WT, *Δh1* and *Δh2* parasites. *Δh1*-infected intestines showed significant reduction of parasite density (*P*< 0.0001, Dunn’s Multiple Comparisons test, N = 50 villi counted per section, 4 (WT, *Δh2*) or 6 (*Δh1*) independent sections counted per strain).

**Fig 7:**
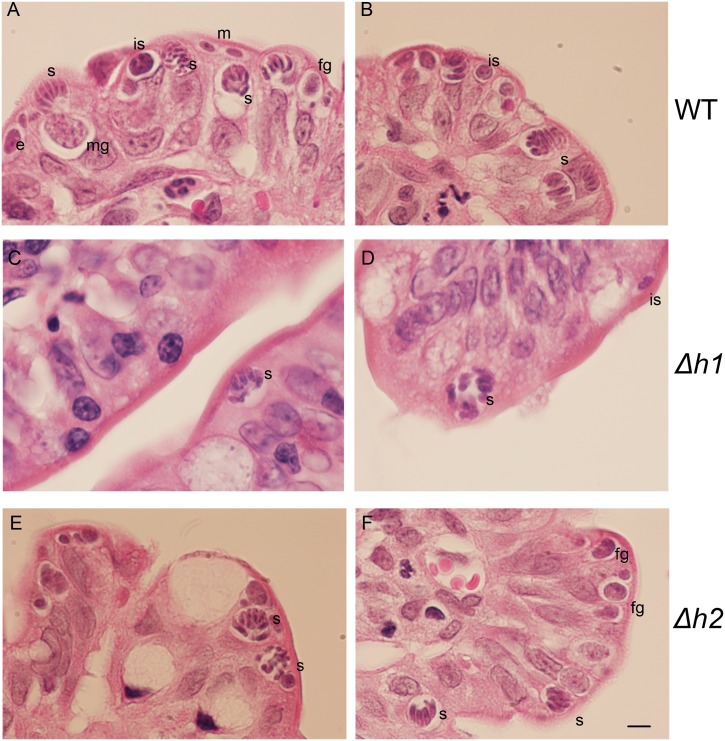
Representative images of hematoxylin and eosin-stained tissue sections of cat intestinal ileum infected with WT (A, B), *Δh1* (C, D), and *Δh2* (E, F) parasites. Multiple stages of the parasite’s sexual cycle can be seen. m: merozoite, is: immature schizont, s: schizont, fg: female gamont, mg: male gamont. Scale bar = 5μm.

**Fig 8:**
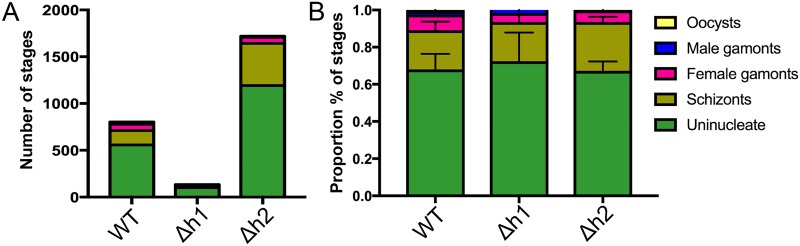
Distribution of parasite sexual developmental stages in cat intestinal ileums infected with WT, *Δh1* or *Δh2* mutant parasites. (A) Pooled counts from four (WT, *Δh2*) or six (*Δh1*) independent intestinal sections from an infected cat are summarized. (B) The relative proportion of each stage observed across the four or six samples are shown +/- SD. No significant differences in the distribution of parasite stages were seen (*P>* 0.9999, Two-way ANOVA).

We reasoned that any defect during asexual expansion in the cat intestine or during the sexual cycle could cause a block that resulted in fewer oocysts being formed. Infection in the cat intestine initially proceeds though asexual expansion, termed A-E forms, which divide by endodyogeny and schizogony, before sexual development commences with the formation of macro and microgamocytes [2]. This process culminates with the exflaggelation of microgametes followed by fertilization of the macrogamete to yield a zygote that matures into an oocyst [2]. To examine the parasite infectivity and development of stages that occur in the cat intestine, we euthanized animals during the initial phase of oocyst shedding and examined tissue sections by conventional histology. In tissue sections from cats infected with the wild type (WT), *Δaah1* (*Δh1*) and *Δaah2* (*Δh2*) parasites taken at 6–7 days post-infection, parasite infection of the intestinal ileum was readily seen (Fig 6A–6C). However, the *Δaah1* parasites showed a significant defect in overall density of infection (Fig 6D). We were readily able to recognize merozoites, schizonts, microgamonts and macrogamonts, indicating that these lines grow well in the gut (Fig 7). Although the density of infection was lower in the *Δaah1* mutant (Fig 8A), the relative distribution of parasite stages was not significantly different (Fig 8B, S4 Table), ruling out the possibility of a defect in any specific stage of parasite sexual development inside the intestinal ileum. Collectively, these findings indicate that *AAH1* plays a role in infection in the cat intestine, and that both *AAH1* and *AAH2* affect the efficiency of oocyst formation *in vivo*, and to a lesser extent the sporulation efficiency, and that these phenotypes are partially penetrant.

## Discussion

Previous studies have suggested that the presence of aromatic amino acid hydroxylase genes *AAH1* and *AAH2* in *T*. *gondii* may be an adaptation for altering host dopamine levels and thereby affecting behavior [20, 22, 23]. However, in prior studies [24] we were not able to replicate the association between *T*. *gondii* infection and elevated dopamine that was seen in mice [41] or in dopaminergic cell lines [22]. Additionally, alternative explanations for the *AAH* genes are provided by studies showing that oocyst walls of *E*. *maxima* [16, 17] contain dityrosine crosslinks, and fluorescence under UV illumination suggests similar modifications exist in *T*. *gondii* oocyst walls [29]. To resolve the potential role of the *T*. *gondii AAH* genes in oocyst formation, we disrupted one or both genes using CRIPSR-based genome editing [33]. Our findings reveal that *AAH2* plays a moderate role, while *AAH1* plays a much stronger role in formation of oocysts during infection in the cat. Additionally, *AAH1* may play a role in parasite survival inside the cat intestinal epithelium as it showed a defect in infectivity even at early stages of merogony and schizogony. It is possible that dityrosine or other L-DOPA derived products produced by these *AAH* genes play a protective role in shielding or cloaking the parasite from the host’s innate immune response in a manner analogous to the role of melanin in *Cryptococcus neoformans* [42], or the *AAH* genes may play an additional role in nutrient availability for the parasite, converting scavenged phenylalanine to tyrosine or vice-versa. Although these findings do not rule out a CNS role for the *AAH* genes, they suggest that one primary function is during infection in the cat intestine, leading to formation of mature oocysts.

Although the *AAH* genes of *T*. *gondii* have been proposed as candidate effectors for the parasite’s ability to manipulate host behavior via manipulating dopamine in the host [23, 25, 41, 43–48], our previous work failed to reproduce the parasite’s described ability to exert effects upon host dopamine levels [24], consistent with other reports [25, 26]. Further, inconsistencies in cat-aversive behavior and other reported behavioral changes including anxiety, activity level, learning, memory, and more, challenge the robustness of this behavioral manipulation [25, 26, 46, 49–53]. Finally, the hypothesis that tissue cysts of brain-resident parasites actively alter host dopamine to exert behavioral control faces exceptional challenge from the observation that parasites defective in their ability to establish lifelong residency in the brain still result in abnormal cat attraction [51]. Additionally, the expression of the *AAH* genes is relatively low in both the lytic and chronic asexual stages [24] and is only upregulated in the sexual stages [30] and mass spectrometry has failed to find evidence of these proteins in tachyzoite or bradyzoite stages but identified them in the oocyst [14]. Hence if the *AAH* gene products are involved in altering dopamine levels in the CNS of infected rodents, they would need to do so based on exceedingly low expression levels, and in a localized region. We are presently examining neurotransmitter levels and behavioral change in mice infected with *AAH* mutants describe here, and such studies could potentially resolve the role of these genes in such pathways.

Because of the high variability in findings regarding the effects of *T*. *gondii* infection on brain neurotransmitters and behaviors, we sought to explore alternate roles for these genes in the parasite life cycle. One obvious candidate would be the contribution of L-DOPA to the formation of protein-protein dityrosine crosslinks in the proteinaceous oocyst wall, analogous to what has been described in *E*. *maxima* [16, 17]. Recently, the oocyst wall proteins *TgOWP1-7*, which are cysteine-rich structural proteins analogous to the *Cryptosporidium* oocyst wall proteins, were characterized and shown to localize to the outer oocyst wall, but not the inner sporocyst walls [19]. Mass spectrometry data also reveal that tyrosine rich proteins are found in oocysts [13, 14], but as yet there is not direct biochemical evidence for dityrosine cross linked proteins in the oocyst wall. However, consistent with the presence of such crosslinks, both the outer oocyst wall and inner sporocyst walls show dityrosine fluorescence, although the signal is significantly brighter in the sporocyst walls. Using the efficiency of CRISPR/Cas9 to direct genetic disruption, we demonstrated that ablation of *AAH1* or both *AAH1* and *AAH2* causes a severe defect in oocyst yield, as well as a maturation defect in the oocysts that do emerge. Parasites ablated for *AAH1* were compromised in replication and development during growth in the cat intestine, and parasites ablated for *AAH2* were able to develop normally within the cat intestine but were compromised in their yield and maturation efficiency after shedding into the environment.

One potential function for the *AAH* genes is in generating modified tyrosine residues (i.e. 3,4 dihydroxyphenylalanine) that are the precursor for dityrosine crosslinks in oocyst wall proteins. This modification is expected to increase oocyst resistance to environmental conditions. The observed decrease in oocyst yield from the *aah* mutants following purification from cat feces is consistent with them being more fragile and prone to loss during the intensive process of osmolar, physical, and chemical treatments that are used during isolation. Although we were able to recover a small number of oocysts from the mutants, they underwent sporulation less efficiently. Since sporulation is associated with increased levels of UV fluorescence, the reduced rate of sporulation in the *aah* mutants is consistent with formation of fewer dityrosine crosslinks. However, some oocysts shed by the mutants were able to undergo sporulation and form oocysts with normal UV fluorescence, although at a much lower total numbers than the wild type. This suggests that if the AAH enzymes normally participate in dityrosine crosslinks, this function can be rescued in the absence of the parasite genes, albeit inefficiently. In this regard, there are at least two other potential sources for 3,4 dihydroxyphenylalanine that serves as a precursor for this reaction: the host cell and the microbiome. Hence, it is possible that salvage from these other sources may enable *T*. *gondii* to generate dityrosine crosslinks at a lower frequency in the absence of *AAH* genes.

Combined with previous findings, our results suggest that *T*. *gondii* builds its oocyst walls using a hybrid strategy combining features of *Cryptosporidium*’s cysteine-cross-linked walls and *Eimeria*’s dityrosine-cross-linked walls. We hypothesize that the proteinaceous part of the outer oocyst wall of *T*. *gondii* is predominantly *Cryptosporidium*-like, composed of *TgOWPs* cross-linked by disulfides. A secondary *Eimeria*-like component of tyrosine-rich proteins cross-linked by dityrosines comprises the proteinaceous inner sporocyst walls in *T*. *gondii* oocysts. In this model, the aromatic amino acid hydroxylases *AAH1* and *AAH2* are expected to catalyze the conversion of tyrosine residues on wall proteins into 3,4 dihydroxyphenylalanine residues for subsequent dityrosine bond formation. The final conversion of these residues into cross-linked proteins is also likely to require a peroxidase, and a putative oxidoreductase that reliably emerges as the most abundant protein in mass spectrometry analyses provides a candidate for this activity [13, 14, 30]. The reduction in infectivity in the *AAH1* mutant suggests that dityrosine or secondary quinones may also play a role as a virulence factor throughout earlier stages of development, analogous to the role of melanin in the neurotropic yeast *Cryptococcus neoformans* [42]. Alternately, the *AAH* genes may be involved in the conversion of phenylalanine to tyrosine to cope with nutrient limitations for growth *in vivo*. To test these models, further studies would be needed to define the localization of the putative tyrosine-rich protein precursors, confirm the presence of dityrosine crosslinks, and investigate the interaction of the *AAH* enzymes with such substrates during sexual stage and oocyst development. However, at present such studies are hindered by the necessity for sexual development to take place in the complex environment of the cat intestine. However, further exploration of these pathways may also be of value for defining attenuated mutants of *T*. *gondii* that are unable to yield infectious oocysts and yet which may induce protective immunity in the cat, thus potentially breaking transmission of the life cycle.

## Supporting information

S1 Table Strains and clones used in this study. (PDF)

S2 Table Plasmids used in this study. (PDF)

S3 Table Primers and plasmids used in this study. (PDF)

S4 Table Number of enteroepithelial stages in HE-stained histological sections of cat ileum. (PDF)
