# Supplementary material for: The aromatic amino acid hydroxylase genes AAH1 and AAH2 in Toxoplasma gondii contribute to transmission in the cat
Source: PLoS Pathog. 2017 Mar 13;13(3):e1006272. doi: 10.1371/journal.ppat.1006272 (PMC5363998; doi:10.1371/journal.ppat.1006272)
Supplement: S1 Table — (PDF) [file ppat.1006272.s001.pdf]

S1 Table Clones used in this study

Clone number	Clone name	Short name	Derived from	Genotype	Source
#1	WT	WT	n/a	ME49 *Δhxgprt::LUC*	Laura Knoll
#2	*Δaah2::HXG*	*Δh2-HXG*	#1	ME49 *Δhxgprt::LUC; Δaah2::HXGPRT*	This paper
#3	*Δaah1*	*Δh1*	#1	ME49 *Δhxgprt::LUC; Δaah1::DHFR-Ts*	This paper
#4	*Δaah2*	*Δh2*	#2	ME49 *Δhxgprt::LUC; Δaah2*	This paper
#5	*Δaah1Δaah2*	*Δh1Δh2*	#3	ME49 *Δhxgprt::LUC; Δaah2; Δaah1::DHFR-Ts*	This paper
#6	*Δaah1-AAH1*	*Δh1-H1*	#5	ME49 *Δhxgprt::LUC; Δaah1::DHFR-Ts/ Δuprt::AAH1*	This paper
#7	*Δaah2::AAH2*	*Δh2-H2*	#4	ME49 *Δhxgprt::LUC; Δaah2::AAH2*	This paper
#8	*Δaah1Δaah2-AAH1*	*Δh1Δh2-H1*	*#5*	ME49 *Δhxgprt::LUC; Δaah2; Δaah1::DHFR-Ts/ Δuprt::AAH1*	This paper
