# Supplementary material for: The aromatic amino acid hydroxylase genes AAH1 and AAH2 in Toxoplasma gondii contribute to transmission in the cat
Source: PLoS Pathog. 2017 Mar 13;13(3):e1006272. doi: 10.1371/journal.ppat.1006272 (PMC5363998; doi:10.1371/journal.ppat.1006272)
Supplement: S2 Table — (PDF) [file ppat.1006272.s002.pdf]

S2 Table Plasmids used in this study

Plasmid name	Use	Source
CRISPR Plasmids		
pSAG1:CAS9,U6:sgUPRT	Single-cutter of UPRT	Shen et al. 2014
pSAG1:CAS9,U6:sgAAH2	Single-cutter of AAH2 in 5' UTR	This paper
pSAG1:CAS9,U6:dgAAH2	Double-cutter of AAH2 in 5' and 3' UTR	This paper
pSAG1:CAS9,U6:sgAAH1	Single-cutter of AAH1 in 5' UTR	This paper
pSAG1:CAS9,U6:dgAAH1	Double-cutter of AAH1 in 5' and 3' UTR	This paper
pSAG1:CAS9,U6:dgHXGPRT	Double-cutter of HXGPRT near 5' and 3' of gene	This paper
AAH Knockouts		
pΔaah2::HXG	Replacement of AAH2 with HXGPRT	Wang et al. 2014
pΔaah2	Replacement of HXGPRT with blank AAH2 5'/3'UTR fusion	Wang et al. 2014
pAAH2	Replacement of HXGPRT with AAH2 cDNA with 5'/3' UTRs	Wang et al. 2014
pΔaah1::DHFR-Ts	Replacement of AAH1 with DHFR-Ts	This paper
pΔuprt::AAH1	Intermediate plasmid to build pΔuprt::AAH1::HXG	This paper
pΔuprt::AAH1::HXG	Replacement of UPRT with AAH1 cDNA with 5'/ 3' UTRs	This paper
