# Supplementary material for: The aromatic amino acid hydroxylase genes AAH1 and AAH2 in Toxoplasma gondii contribute to transmission in the cat
Source: PLoS Pathog. 2017 Mar 13;13(3):e1006272. doi: 10.1371/journal.ppat.1006272 (PMC5363998; doi:10.1371/journal.ppat.1006272)
Supplement: S3 Table — (PDF) [file ppat.1006272.s003.pdf]

S3 Table Primers and plasmids used in this study

Primer Category	Primer Name	Primer Sequence	Use	Used for plasmid	Source
AAH2 Knockout, Cleanup, Complementation					
	Z22 TgAaaH2 KO fwd cassette fwd primer attB4	5'GGGGACAACTTTGTATAGAAAAGTTGCAATTGTGCTTACGCAAGCTCGGC3'	*AAH2* Knockout	*pΔaah2::HXG*	Wang et al. 2014
	Z23 TgAaaH2 KO fwd cassette rev primer attB1r	5'GGGGACTGCTTTTTTGTACAAACTTGCAAAGCATTAGGCAAGGCTGCAGG3'	*AAH2* Knockout	*pΔaah2::HXG*	Wang et al. 2014
	Z24 TgAaaH2 KO rev cassette fwd primer attB2r	5'GGGGACAGCTTTCTTGTACAAAGTGGAAAGTCGTCGTCCCCTGCGATTCG3'	*AAH2* Knockout	*pΔaah2::HXG*	Wang et al. 2014
	Z25 TgAaaH2 KO rev cassette rev primer attB3	5'GGGGACAACTTTGTATAATAAAGTTGGGTGAACATGCGGAGATGAAAGACCC3'	*AAH2* Knockout	*pΔaah2::HXG*	Wang et al. 2014
	Z22 TgAaaH2 KO fwd cassette fwd primer attB4	5'GGGGACAACTTTGTATAGAAAAGTTGCAATTGTGCTTACGCAAGCTCGGC3'	*AAH2* Cleanup	*pΔaah2*	Wang et al. 2014
	Z69 TgAaaH2 Clean KO pri1 rev	5'CGAATCGCAGGGGACGACGACTTTCAAAGCATTAGGCAAGGCTGCAGG3'	*AAH2* Cleanup	*pΔaah3*	Wang et al. 2014
	Z70 TgAaaH2 Clean KO pri2 fwd	5'AAAGTCGTCGTCCCCTGCGATTCG3'	*AAH2* Cleanup	*pΔaah4*	Wang et al. 2014
	Z71 TgAaaH2 Clean KO pri2 rev attB1r	5'GGGGACTGCTTTTTTGTACAAACTTGGGTGAACATGCGGAGATGAAAGACCC3'	*AAH2* Cleanup	*pΔaah5*	Wang et al. 2014
	Z60 TgAaaH2 Complement pri1 fwd attB4	5'GGGGACAACTTTGTATAGAAAAGTTGTGCGAGCACGTTATCAAATCTTGG3'	*AAH2* Complement	*pAAH2*	Wang et al. 2014
	Z72 TgAaaH2 Complement pri1 rev	5'GCACGCAGCGCGGTGGATAGAGACATCGATTTAGAATGCGTTACGGTGATCGGAG3'	*AAH2* Complement	*pAAH2*	Wang et al. 2014
	Z66 TgAAH1/2 cDNA Fwd	5'ATGTCTCTATCCACCGCGCTGCG3'	*AAH2* Complement	*pAAH2*	Wang et al. 2014
	Z73 TgAAH2 Complement pri3 fwd	5'GCCTCCTGTCTCCCTCAAGATCTAGACCAGACACTGTATCTCTACTTTGCATTCG3'	*AAH2* Complement	*pAAH2*	Wang et al. 2014
	Z65 TgAAH2 Complement pri3 rev attB1r	5'GGGGACTGCTTTTTTGTACAAACTTGCCTTCGCTTGACTGTATCTGCTTGC3'	*AAH2* Complement	*pAAH2*	Wang et al. 2014
	A14 HXGPRT 5' Rev screening primer	5'CATACTAGTTTCAATGATCCCCCTCCACCGCGG3'	*HXGPRT* Screening		Wang et al. 2014
	A26 HXGPRT 3' Fwd screening primer	5'GATGAATTCCAGCACGAAACCTTGCATTCAAAC3'	*HXGPRT* Screening		Wang et al. 2014
	Z28 AAH1/2 internal 5' primer	5'GGGGACAACTTTGTATAGAAAAGTTGCAGGATTGCTCTCCTCACCAGGGG3'	*Δaah2* Screening		Wang et al. 2014
	Z68 AAH2 3' rev	5'CTAGATCTTGAGGGAGACAGGAGGCATATG3'	*Δaah2* Screening		Wang et al. 2014
AAH1 Knockout & Complementation					
	X67 AAH1 Knockout 5' ' fwd pUC19 homology	5'TTGTAAAACGACGGCCAGTGCGATGTTCATGCAGTTGGTGTCTC3'	*AAH1* Knockout	*pΔaah1::DHFR-Ts*	This paper
	X68 AAH1 Knockout 5' ' rev DHFR homology	5'GCTATACGAAGTTATGGATCCATCGAGGGACAGACTGCGGTACATCG3'	*AAH1* Knockout	*pΔaah1::DHFR-Ts*	This paper
	X69 AAH1 Knockout 3' ' fwd DHFR homology	5'ATTATACGAAGTTATCGATCGTAGCTTGGTGTCTCCCACTATCTTCTGC3'	*AAH1* Knockout	*pΔaah1::DHFR-Ts*	This paper
	X70 AAH1 Knockout 3' ' rev pUC19 homology	5'GATTACGCCAAGCTTGCATGAGACACTCCGACAGTCAGGAATCC3'	*AAH1* Knockout	*pΔaah1::DHFR-Ts*	This paper
	E06 DHFR-Ts Construct 5'	5'GATGGATCCATAACTTCGTATAGCATACATTATACGAAGTTATCAGCACGAAACCTTGCATTCAAACCCGC	*AAH1* Knockout	*pΔaah1::DHFR-Ts*	This paper
	E07 DHFR-Ts Construct 3'	5'CTACGATCGATAACTTCGTATAATGTATGCTATACGAAGTTATATCCTGCAAGTGCATAGAAGGAAAGTT	*AAH1* Knockout	*pΔaah1::DHFR-Ts*	This paper
	X44 UPRT 5' UTR fwd pUC19 homology	5'TTGTAAAACGACGGCCAGTGGGAACACCATATTTGCATTGCACGCTTG3'	*AAH1* Knockout	*pΔuprt::AAH1*	This paper
	X48 UPRT 5' UTR rev +loxP X25 homology	5'GACCTCCATTTTGTCGGAATATAACTTCGTATAATGTATGCTATACGAAGTTATTCTAGAGATAACGGTTC	*AAH1* Knockout	*pΔuprt::AAH1*	This paper
	X25 AAH1 5' UTR fwd	5'ATTCCGACAAAATGGAGGTCGCGAGAGAAGAAACAACTCCGTC3'	*AAH1* Knockout	*pΔuprt::AAH1*	This paper
	Z83 AAH1 5' UTR rev	5'CGCAGCGCGGTGGATAGAGACATCGATTTAGAATACATTACGGTTACCCGAGAC3'	*AAH1* Knockout	*pΔuprt::AAH1*	This paper
	Z66 AAH1/AAH2 cDNA 5' fwd	5'ATGTCTCTATCCACCGCGCTGCG3'	*AAH1* Knockout	*pΔuprt::AAH1*	Wang et al. 2014
	Z67 AAH1 cDNA 3' rev	5'CTAGAACCTGAGGGAAACGGGCAGAGCTCG3'	*AAH1* Knockout	*pΔuprt::AAH1*	Wang et al. 2014
	Z84 AAH1 3' UTR fwd	5'CGAGCTCTGCCCGTTTCCCTCAGGTTCTAGGTTACAGACCGACTCTACATTTCGCAGTTG3'	*AAH1* Knockout	*pΔuprt::AAH1*	This paper
	X19 AAH1 3' UTR rev	5'GTTTCGTGCTGGAATTCATCGATGGCAACGAACAAGTGATGTTGC3'	*AAH1* Knockout	*pΔuprt::AAH1*	This paper
	X49 UPRT 3' UTR fwd loxP X19 homology	5'GATGAATTCCAGCACGAAACATAACTTCGTATAGCATACATTATACGAAGTTATGGTATATCTTTGGTCGC	*AAH1* Knockout	*pΔuprt::AAH1*	This paper
	X47 UPRT 3' UTR rev pUC19 homology	5'GATTACGCCAAGCTTGCATGTGACCGCAGAAGACACCTGCTATCC3'	*AAH1* Knockout	*pΔuprt::AAH1*	This paper
	X58 HXG insertion into pΔuprt::AAH1 fwd	5'GAAGAGAAGGAAAAAAATTGGATGAATTCCAGCACGAAACCTTGC3'	*AAH1* Knockout	*pΔuprt::AAH1::HXG*	This paper
	X59 HXG insertion into pΔuprt::AAH1 rev	5'TTCTCTGCGCGGTGGCCTTGCATACTAGTTTCAATGATCCCCCTC3'	*AAH1* Knockout	*pΔuprt::AAH1::HXG*	This paper
AAH1/AAH2 Knockout Screening					
	Y46 TgTubulin fwd	5'GGGGACAGCTTTCTTGTACAAAGTGGAAGGCGTAACTCAGGACGGCTTG3'	Screening		This paper
	Y47 TgTubulin rev	5'CGCAGCGCGGTGGATAGAGACATAAAAGGGAATTCAAGAAAAAATGCCAACGAGTAG3'	Screening		This paper
	Z28 AAH1/ AAH2 fwd (exon)	5'GGGGACAACTTTGTATAGAAAAGTTGCAGGATTGCTCTCCTCACCAGGGG3'	Screening		Wang et al. 2014
	Z67 AAH1 3' rev	5'CTAGAACCTGAGGGAAACGGGCAGAGCTCG3'	Screening		Wang et al. 2014
	Z68 AAH2 3' rev	5'CTAGATCTTGAGGGAGACAGGAGGCATATG3'	Screening		Wang et al. 2014
AAH1/ AAH2 Locus Screening					
	Z28 AAH1/ AAH2 fwd (exon)	5'GGGGACAACTTTGTATAGAAAAGTTGCAGGATTGCTCTCCTCACCAGGGG3'	PCR Amplification		Wang et al. 2014
	Z65 AAH1/AAH2 rev (3' UTR homologous region)	5'GGGGACTGCTTTTTTGTACAAACTTGCCTTCGCTTGACTGTATCTGCTTGC3'	PCR Amplification		Wang et al. 2014
	Z28 AAH1/ AAH2 fwd (exon)	5'GGGGACAACTTTGTATAGAAAAGTTGCAGGATTGCTCTCCTCACCAGGGG3'	Sanger Sequencing		Wang et al. 2014
CRISPR Plasmid Mutagenesis					
	X13 CRISPR HXGPRT Targeting Site 1	5'CACATCATTTGCATCCTGAAGTTTTAGAGCTAGAAATAGC3'	5' sgRNA for Q5 mutagenesis	*pSAG1:CAS9,U6:dgAAH1*	This Paper
	X14 CRISPR AAH1 Targeting Site 1 (5' UTR)	5'TCCCTTATTTGCTTCTGTACGTTTTAGAGCTAGAAATAGC3'	5' sgRNA for Q5 mutagenesis	*pSAG1:CAS9,U6:dgAAH1*	This Paper
	X15 CRISPR AAH1 Targeting Site 2 (3' UTR)	5'TATCAGAGAGCACAGCAACGGTTTTAGAGCTAGAAATAGC3'	5' sgRNA for Q5 mutagenesis	*pSAG1:CAS9,U6:dgAAH1*	This Paper
	X16 CRISPR AAH2 Targeting Site 1 (5' UTR)	5'AATTCTCAATTGCAGCACACGTTTTAGAGCTAGAAATAGC3'	5' sgRNA for Q5 mutagenesis	*pSAG1:CAS9,U6:dgAAH2*	This Paper
	X17 CRISPR AAH2 Targeting Site 2 (3' UTR)	5'GTTACGTTTCTTTCTCTCGTGTTTTAGAGCTAGAAATAGC3'	5' sgRNA for Q5 mutagenesis	*pSAG1:CAS9,U6:dgAAH2*	This Paper
	X50 CRISPR HXGPRT Targeting Site 2	5'TGTTGTTGCACTCCTAATGAGTTTTAGAGCTAGAAATAGC3'	5' sgRNA for Q5 mutagenesis	*pSAG1:CAS9,U6:dgHXGPRT*	This Paper
	3'-Rop18-gRNA rev	5' AACTTGACATCCCCATTTAC3'	3' Primer for Q5 mutagenesis	all CAS9 plasmids	This Paper
	sgRNA cassette KpnI fwd	5'CGAATTGGGTACCCAAGTAAGCAGAAGCACGCTG3'	Amplifying 2ndary sgRNA	all CAS9 doublecutter plasmids	Behnke et al. 2015*
	sgRNA cassette XhoI rev	5'TCGACCTCGAGAATTAACCCTCACTAAAGG3'	Amplifying 2ndary sgRNA	all CAS9 doublecutter plasmids	Behnke et al. 2015*

* Behnke MS, Khan A, Lauron EJ, Jimah JR, Wang Q, et al. (2015) Rhoptry Proteins ROP5 and ROP18 Are Major Murine Virulence Factors in Genetically Divergent South American Strains of Toxoplasma gondii. PLOS Genetics 11(8): e1005434. doi: 10.1371/journal.pgen.1005434
