# Supplementary material for: The aromatic amino acid hydroxylase genes AAH1 and AAH2 in Toxoplasma gondii contribute to transmission in the cat
Source: PLoS Pathog. 2017 Mar 13;13(3):e1006272. doi: 10.1371/journal.ppat.1006272 (PMC5363998; doi:10.1371/journal.ppat.1006272)
Supplement: S4 Table — (PDF) [file ppat.1006272.s004.pdf]

S4 Table Number of enteroepithelial stages

Parasite Line	Sample number	Uninucleate	Schizonts	Female gamonts	Male gamonts	Oocysts
WT	1	150	52	37	3	0
	2	343	81	25	14	3
	3	20	10	4	0	1
	4	45	10	1	2	1
	**Total**	**558**	**153**	**67**	**19**	**5**
Δh1	1	26	5	0	0	0
	2	47	16	0	0	0
	3	11	3	1	0	0
	4	8	1	0	0	0
	5	6	1	2	0	0
	6	3	3	0	1	0
	**Total**	**101**	**29**	**3**	**1**	**0**
Δh2	1	170	62	25	4	0
	2	74	31	12	2	0
	3	313	154	21	3	0
	4	636	203	7	2	0
	**Total**	**1193**	**450**	**65**	**11**	**0**
